# STRUCTURAL DIFFERENCES IN HEALTHY YOUNG AND OLD HUMAN MUSCLE CELLS: APPLYING DEEP LEARNING TO VOLUMETRIC SEM IMAGES

**DOI:** 10.1093/geroni/igad104.0587

**Published:** 2023-12-21

**Authors:** Lisa Hartnell

**Affiliations:** National Institute on Aging, National Institutes of Health, Baltimore, Maryland, United States

## Abstract

Several studies have shown that muscle strength and mitochondrial function degrade in individuals over time as a function of aging and/or disease. To better understand this process, we examined the relationship between structure and function of muscle in healthy young, healthy old and frail old participants in the Genetic and Epigenetic Signatures of Translational Aging Laboratory Testing (GESTALT) study. Muscle was examined at the level of the mitochondrial reticulum and sarcomere, the basic contractile units of muscle, using volumetric Scanning Electron Microscopy (SEM) imaging. Volumetric SEM imaging using Focused Ion Beam (FIB)-SEM yields isotropic volumes enabling analysis at near membrane resolution and is ideal for mapping the mitochondrial reticulum and structural components of the sarcomere in 3D. The mitochondrial reticulum is a highly organized continuum of individual mitochondria that distribute and provide energy for contraction throughout muscle. We will show our approach to apply deep learning segmentation to volumes of gastrocnemius muscle ranging from 10-20 micrometers cubed collected from young (20-29y) and old (75-89y) participants in GESTALT to quantify differences in the reticulum including fragmentation and degeneration, as well as distribution and number of mitochondria. Preliminary findings suggest a difference with increasing age and more marked structural differences in both sarcomeres and mitochondria associated with frailty. Future work aims to increase the number of individuals sampled and extend the analysis to muscle from middle-aged individuals using biological markers and tests that indicate a functional aging phenotype.

